# 'Careful goodbye at the door': is there role for antimicrobial stewardship interventions for antimicrobial therapy prescribed on hospital discharge?

**DOI:** 10.1186/s12879-018-3147-0

**Published:** 2018-05-16

**Authors:** R. Chavada, J. Davey, L. O’Connor, D. Tong

**Affiliations:** 1 Department of Microbiology and Infectious Diseases, NSW Health Pathology, Gosford, NSW Australia; 2 Division of Medicine, Central Coast Local Health District, Wyong, NSW Australia; 3 Pharmacy Department, Central Coast Local Health District, Gosford, NSW Australia

**Keywords:** Antimicrobial stewardship, Interventions, Hospital discharge, Antimicrobial prescriptions

## Abstract

**Background:**

Antimicrobial stewardship (AMS) interventions largely target inpatient antimicrobial prescribing. Literature on appropriateness of antimicrobials prescribed at the interface between hospital and the community is minimal. This study was designed to assess the appropriateness of antimicrobials prescribed on hospital discharge and evaluate the impact of AMS interventions.

**Methods:**

Patients with discharge medications processed by the pharmacy were identified using a computerized pharmacy medication tracker over a four week period. The antimicrobials prescribed on discharge were assessed independently for appropriateness of antimicrobial choice, dose, frequency and duration. Data on various AMS interventions was collected. Univariate followed by multivariate logistic regression (MVLR) analysis was performed using SPSS V 23 (IBM, California).

**Results:**

A total of 892 discharge prescriptions were processed by the pharmacy department, 236 of which contained antibiotic prescriptions. Of these, 74% were appropriate for antimicrobial choice, 64% for dose, 64% for frequency and 21% for duration. In particular, 71% of patients received a course in excess of Therapeutic Guidelines-Australia(TG-A) recommended length of treatment. On univariate analysis, discharge antimicrobial prescriptions were more likely to be appropriate for antimicrobial choice, frequency and duration; appropriate microbiological specimens were more likely to be taken and targeted therapy more likely to be given when the AMS team was involved. On MVLR, appropriateness with antimicrobial dosing frequency [OR 5.6(1.9–19.2)], microbiological specimens [OR 4.3(1.6–11.6)] and receipt of targeted therapy [OR 2.8(1.8–6.2)] with AMS involvement remained significant.

**Conclusions:**

A large discrepancy exists between antimicrobial regimens prescribed on hospital discharge and those recommended in consensus guidelines, particularly concerning duration of treatment. While AMS interventions are well established for improving antimicrobial prescribing in hospital inpatients, the hospital-community interface remains a challenge in terms of antimicrobial prescribing and exposes patients to potential harm. There is a clear need for AMS interventions to extend to antimicrobial therapy prescribed on discharge.

## Background

Antimicrobial resistance (AMR) is a serious global health concern with significant clinical and economic sequelae. The incidence of AMR is rapidly increasing around the world, and infections caused by multi-resistant organisms (MROs) are associated with higher incidences of morbidity, mortality, and prolonged hospital admission [1]. Selective pressure of antimicrobial usage on microorganisms is the main driver of the emergence of AMR, therefore considerable focus has been placed on ensuring the judicious use of antimicrobials. Not only does this have implications for the patient who is infected with MROs, but also to the spread of such organisms in healthcare facilities and community at large [2]. For this reason, antimicrobial stewardship (AMS) programs, which aim to rationalize and reduce antimicrobial use in healthcare facilities, are increasingly being promoted and mandated. There are various elements of AMS programs, including pre-prescription authorization, post prescription review, regular ward rounds for review of antimicrobial use, education of prescribers and regular audits with feedback [3].

The majority of these interventions focus on improving antimicrobial prescribing for hospital inpatients, with limited evidence of such activities being performed or providing benefit for patients being discharged from hospital. As such, a recent study noted that 65% of antimicrobial treatment was being completed after hospital discharge, with more than half of these prescriptions found to be inappropriate [4, 5] . The aim of the study was to assess the appropriateness of antimicrobials prescribed on hospital discharge in accordance with the Australian *Therapeutic Guidelines: Antibiotic* (TG-A) and evaluate the impact of AMS interventions on the appropriateness of therapy upon patient discharge [6].

## Methods

### Study setting

Wyong Hospital is a regional metropolitan hospital on the Central Coast of New South Wales, Australia. It is a 300 bed teaching facility with acute medical and surgical care supported by a busy emergency department and intensive care unit. Due to the demographics of the population in the region, geriatric inpatients constitute a substantial proportion of hospitalized patients. A pharmacy department is located on site and facilitates the review and supply of medication prescriptions for inpatients, outpatients and patients being discharged from hospital. As per the hospital policy, prescribed courses of antimicrobials are supplied to patients upon hospital discharge at no cost by the pharmacy department. The AMS team at the hospital consists of an Infectious Diseases (ID) physician and a dedicated AMS pharmacist. AMS ward rounds are performed twice weekly on a regular basis with review of antimicrobial therapy. Additional telephone ID advice is provided on an ad hoc consultation basis, antimicrobial restriction with pre-prescription authorization is also enforced for broad spectrum antimicrobials [7].

### Study design

**Fig. 1:**
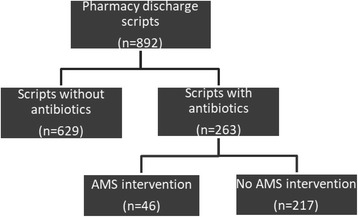
Overview of the antimicrobial discharge study

Since the impact of AMS interventions on discharge antimicrobials was not known, the AMS team formulated a strategy of providing discharge advice in patient electronic medical records as part of routine inpatient AMS interventions. This advice consisted of an individualized plan for continued management of an infectious disease upon hospital discharge, and included appropriate investigations to be performed such as repeat radiology, directed antimicrobial therapy according to microbiological testing, side effects of antimicrobials for prescribers and patients to be aware of, and dose and total duration of therapy. A retrospective audit was performed on all patients prescribed one or more antimicrobials on hospital discharge over this period.. Patients with discharge medications processed by the pharmacy department were identified using a computerized pharmacy medication tracker over a 4 week period (March 2016) and any interventions performed by the AMS team were noted. (Fig. 1) Outcomes of patients who received AMS advice or review on their antimicrobial therapy were compared with those who did not. Due to limitations in our current resources, not all patients who were prescribed antimicrobials were reviewed by the AMS team. AMS team intervention consisted of review of patients medical records, medication chart, pathology and microbiology results and direct discussion or consultation regarding antimicrobial choice, dose, duration, and frequency to the treating clinicians.

Our hospital uses a ‘traffic light’ restriction system for prescribing antimicrobial drugs. The use of ‘green’ antimicrobials is unrestricted and not routinely monitored, while ‘orange’ and ‘red’ antimicrobials are restricted, with ‘orange’ antimicrobials requiring a post-prescription review by the AMS team within 48 h and ‘red’ antimicrobials requiring pre-prescription authorization by an ID physician. ‘Orange’ and ‘red’ antimicrobials are monitored and restricted to a greater extent as these agents are broader spectrum, have more side effects, require therapeutic drug monitoring and/or are more costly [7].

The appropriateness of prescriptions with regard to antimicrobial choice, dose, frequency and duration was assessed by an ID specialist and ID pharmacist based on standard recommendations in TG-A.^6^ For indications not covered by these guidelines, an assessment of appropriateness was made independently by an ID specialist and ID pharmacist. These were based on other specialty society, international or other hospital guidelines.

Readmission to hospital was defined by readmission to the hospital for any reason within 28 days or within 28 days for management of same (index episode) infection patients were initially treated for.

AMS programs and associated activities are mandatory as a part of hospital accreditation by the Australian Commission on Safety and Quality in Health care (ACSQHC) [8]. Audit of antimicrobial use and feedback to prescribers are routine AMS activities in our hospital. The information collected was considered to be a part of routine and ongoing AMS activities, and this study was performed as a quality improvement project (QI 0118-003C).

### Statistical analysis

The χ^2^ test or Fisher’s exact test was used to analyse the categorical data where appropriate. *P* values ≤0.05 were considered statistically significant. Variables with a univariate association *p*-value less than 0.2 were considered for inclusion in a multivariate logistic regression model. Multivariable stepwise backward logistic regression analysis was also done after adjusting for various clinical specialties as potential confounders. Statistical analysis was performed using IBM SPSS Statistics 22.0 software.

## Results

**Fig. 2:**
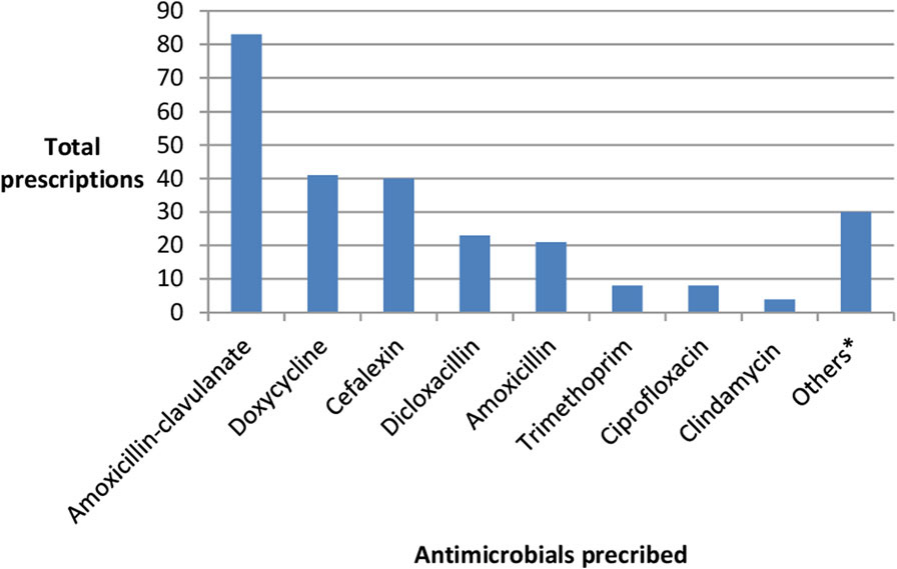
Antimicrobials prescribed on discharge, others are miscellaneous antimicrobials such as cefuroxime, metronidazole, azithromycin, trimethoprim-sulphamethaxazole, rifampicin, fusidic acid, fluconazole and valciclovir

Over the 4 week period a total of 892 discharge prescriptions were processed by the pharmacy department, 263 of which contained antibiotic prescriptions [Fig. 1]. Of these, 74% (*n* = 215) were appropriate for antimicrobial choice, 64% (*n* = 188) for dose, 64% (n = 188) for frequency and 21% (*n* = 62) for duration. In particular, 71% of patients received a course in excess of TG-A length of therapy. The median excess duration was 2 days (range − 6 to 19 days). The negative days in the range indicate a shorter duration of antibiotic therapy received compared with recommended guidelines. Appropriate microbiological specimens were taken in 50% of cases and directed therapy based on susceptibility testing was given in 18% (*n* = 53) of cases. A subspecialty breakdown revealed that the most number of restricted antimicrobials were used by cardiology, with antimicrobials such as vancomycin and extended duration gentamicin being used as directed therapy for infective endocarditis. This was followed by general medicine with 7.4% of total restricted antimicrobial use, which mainly comprised of ciprofloxacin being used to treat chronic respiratory infections [Table 1]. Amoxicillin-clavulanate (*n* = 85) was the most commonly prescribed antimicrobial on discharge, followed equally by cefalexin (*n* = 40) and doxycycline (n = 40) [Fig. 2].

**Table 1:** Antimicrobials prescribed by clinical specialties

Clinical Specialities	Restricted antimicrobial	Unrestricted antimicrobial	Percent(%) use of restricted agents
Respiratory	2	32	6
General medicine	8	105	7.4
Geriatrics	1	16	6
Surgery ^a^	4	60	6.6
Cardiology	4	9	44
Urology	0	8	0
Total	19	230	

^a^includes general, colorectal and orthopaedic

The univariate analysis for restricted and unrestricted antimicrobials prescribed based on appropriateness of choice, duration and frequency is shown in Table 2. On univariate analysis restricted antimicrobials were more likely to be appropriate for duration (*p* = 0.019), dosing frequency (*p* = 0.025) and dose (*p* = 0.028) compared with unrestricted antimicrobials. Fluroquinolones constituted 9 of 23 (43%) of restricted antimicrobials prescribed. The appropriateness of antimicrobial choice did not differ statistically between the restricted and unrestricted group. On multivariate analysis, the duration [OR 2.6(1.1–6.4, *p* = 0.031)] and dosing frequency [OR 4.4(1.0–19.7, *p* = 0.047)] of restricted antimicrobials was more likely to be appropriate on discharge than unrestricted antimicrobials.

**Table 2:** Univariate and multivariate analysis of restricted antimicrobial prescribed in the study

Antimicrobial prescription parameter	Restricted antimicrobial (*n* = 23) n(%)	Unrestricted antimicrobial (*n* = 240) n(%)	Univariate analysis (*p* value)	Multivariate analysis (Odds ratio with 95%CI, *p* value)
Choice	22(95)	193(80)	0.072	
Duration	10(43)	52(21)	0.019	OR 2.6(1.1–6.4, p = 0.031)
Dose	21(91)	167(70)	0.028	
Frequency	21(91)	166(69)	0.025	OR 4.4(1.0–19.7, p = 0.047)

The univariate analysis for involvement of the AMS team and appropriateness of discharge antimicrobial prescriptions, microbiological specimens sent, directed therapy and hospital readmission within 28 days are shown in Table 3 which demonstrate that discharge antimicrobial prescriptions were more likely to be appropriate for dosing (*p* = 0.073), appropriate microbiology specimen sent (*p* = 0.001), targeted therapy (p = 0.001), antimicrobial choice (*p* = 0.006), and duration (*p* = 0.023) when the AMS team were involved. There was no significant difference in hospital readmission rates between those who received AMS interventions and those who did not. *Clostridium difficile* infection (CDI) secondary to antimicrobial therapy was only seen in one patient, who had a skin and soft tissue infection treated with cephazolin and de-escalated to cefalexin. Three patients, one of whom was in the AMS intervention arm, were readmitted to hospital within 28 days for management of the same infection they were initially treated for, while all other readmissions were for unrelated reasons. All three patients had a chronic respiratory condition. On multivariate logistic regression analysis, appropriateness of dosing frequency [OR 5.6(1.9–19.2), *p* = 0.006], microbiological specimens [OR 4.3(1.6–11.6), *p* = 0.004] and receipt of directed therapy [OR 2.8(1.8–6.2), *p* = 0.008], when the AMS team was involved, remained statistically significant.

**Table 3:** Univariate and multivariate analysis showing involvement of the AMS team in discharge antimicrobial prescriptions

Antimicrobial Prescription parameter	AMS involved (*n* = 46) n(%)	AMS not involved (*n* = 217) n(%)	*p* value	Multivariate analysis (Odds ratio with 95%CI, p value)
Appropriate antimicrobial choice	44(95)	171(78)	0.006	
Appropriate duration	17(37)	45(21)	0.023	
Appropriate dose	38(82)	150(69)	0.073	
Appropriate frequency	43(93)	144(66)	0.001	OR 5.6(1.9–19.2,p = 0.006)
Appropriate microbiology specimen	40(86)	104(48)	0.001	OR 4.3(1.6–11.6,p = 0.004)
Directed therapy	23(50)	30(14)	0.001	OR 2.8(1.8–6.2,p = 0.008)
Readmission^a^	12(26)	36(16)	0.14	

^a^Hospital readmission definition as per methods section

## Discussion

While AMS interventions are well established and increasingly mandatory in many countries for optimizing antimicrobial prescribing in hospital inpatients, this is the first study to our knowledge to investigate the impact of such interventions on the appropriateness of antimicrobial therapy prescribed across a broad range of infection which were treated in hospitals and who had antimicrobials prescribed upon hospital discharge. Our finding of significant levels of inappropriate antimicrobial prescriptions in this setting is consistent with other studies which identified the similar issue of with high volume of inappropriate prescriptions [4, 9]. Given that most antimicrobial courses are completed after hospital discharge, ensuring appropriateness of therapy should be a priority area for AMS programs [10, 11]. The recent position paper from the Infectious Diseases Society of America (IDSA) outlines various strategies for implementing AMS in hospital settings but lacks information on AMS interventions upon hospital discharge [5]. A recent inventory survey by the European Society of Clinical Microbiology and Infectious Diseases (ESCMID) Study Group for Antimicrobial StewardshiP (ESGAP) reported that measurable quality (appropriateness) and quantity (volume or cost) indicators in antimicrobial prescribing are lacking in several countries and that development of such indicators would lead to more appropriate prescribing [12]. Our study assessed for quality (appropriateness) and quantity (volume) of antimicrobials prescribed upon hospital discharge, a domain where there is lack of information.

Excessive antimicrobial treatment duration is associated with an increased risk of antimicrobial resistance and CDI, as well as unnecessary healthcare costs [4]. In two previous studies it was found that an excessive treatment course was prescribed in 33 and 55% of hospital discharge antimicrobial prescriptions respectively [4, 9]. 71% of patients in our study received a course in excess of guideline-recommended length of treatment. This was also found in another study by Hoover in a US medical center [13]. We hypothesize this may be because doctors may not account for the intravenous and oral antimicrobial therapy already received by the patient in hospital, and simply prescribe a standard 5- or 7-day ‘course’ upon discharge. This may be due to time pressures experienced by junior medical officers when prescribing discharge medications in a busy hospital, or based upon advice from a more senior member of the treating team. Although treatment guidelines with specific treatment duration advice are widely available in our institution, they are usually referred to at the commencement of therapy, rather than toward the end to guide duration of therapy. Prescriber behavioral factors such as fear of treatment failure or readmission and habit may also contribute. In a survey performed recently at our hospital, we found that experience-based prescribing was still occurring, although with ongoing education and feedback such prescribing habits were declining overall [7]. While the duration of treatment was more likely to be appropriate if the AMS team were involved, other practical interventions such as prescriber education, prospective audit and feedback specifically for discharge antimicrobials and the introduction of electronic prescribing and flagging may also help to improve prescribing in this particular area.

While the distinction between restricted and unrestricted antimicrobial agents is not absolutely rigorous, in our hospital and generally throughout most Australian hospitals this distinction is maintained for the purpose of AMS. We think that certainly fluroquinolone use in hospitalized patients and upon discharge clearly needs monitoring and assessment for appropriateness. Studies in the literature have highlighted the impact of fluroquinolone restriction on overall reduction of its use and CDI rates [14, 15]. Given both pre-prescription authorization and post-prescription review are in place as part of the AMS program in our hospital, it is not surprising but nonetheless pleasing that the use (duration, dosing frequency and dose) of restricted antimicrobials was more appropriate than unrestricted antimicrobials. Similarly, observing the benefits of AMS involvement on improved outcomes such as appropriate microbiological specimens being taken and receipt of directed therapy are also not surprising. Other studies with pharmacist-led initiatives have shown similar results and improved overall appropriateness of antimicrobial therapy [16, 17].

As antimicrobials are so commonly prescribed on hospital discharge, it would be prudent to identify the patients, indications and/or antimicrobials at highest risk of harm from inappropriate therapy, as well as to utilize novel methods to extend the coverage of the existing AMS service. For example, our hospital will soon be implementing electronic prescribing and medication management. Strategies suggested in the literature include implementation of an AMS model following patients discharged on antimicrobial therapy with pending culture results and subsequent modification of antimicrobial therapy, a pharmacy-based triage algorithm for pneumonia, mandatory ID consultation of intended community-based parenteral antimicrobial therapy and a checklist framework for pediatric patients [18–20]. We also envisage the introduction of other strategies such as training and upskilling of clinical pharmacists who already perform discharge medication reconciliation for critical review of discharge antimicrobial therapy, building algorithms into the impending electronic prescribing program to identify and flag inpatient antimicrobial courses and targeting patients being discharged to residential aged care facilities. However, further studies are required to evaluate the effectiveness and associated workload of these strategies.

Our study has several limitations. First, it involved a single metropolitan hospital in Australia with a significant geriatric patient base, so our findings may not be generalizable to other settings. Second, the study was performed retrospectively, and relied on the accuracy and completeness of electronic medical records for assessment of appropriateness of antimicrobial therapy prescribed on discharge as well as documentation of intervention by the AMS team. Third, the study was performed over a period of 4 weeks so the antimicrobial prescriptions observed may not be reflective of the general prescribing trends throughout the year. Fourth, discharge prescriptions from the emergency department of our hospital are often provided on outpatient prescription forms and are given directly to the patient to purchase from community pharmacies, thus these prescriptions would not have been captured using the pharmacy department discharge medication tracker in our study.

## Conclusion

In summary, the hospital-community interface is a concerning gap in existing AMS programs. All aspects of discharge antimicrobial prescriptions have the potential for improvement, particularly the duration of treatment. AMS interventions in this setting led to improved compliance with the Australian *Therapeutic Guidelines: Antibiotic*. We think that antimicrobial prescribing at the transition of care between hospital and the community is an important and neglected opportunity that can be improved by targeted AMS interventions.

## Abbreviations

AMR: antimicrobial resistance
AMS: Antimicrobial stewardship
MRO: Multiresistant organism
TG-A: Therapeutic guidelines Australia
